# Beneficial effects of manually assisted chiropractic adjusting instrument in a rabbit model of osteoarthritis

**DOI:** 10.1038/s41598-020-70219-3

**Published:** 2020-08-06

**Authors:** F. M. Conesa-Buendía, A. Mediero, R. Fujikawa, P. Esbrit, F. Mulero, I. Mahillo-Fernández, Arantxa Ortega-De Mues

**Affiliations:** 1 Bone and Joint Research Unit, Institute of Health Research (IIS-Fundación Jiménez Díaz), Madrid, Spain; 2 Madrid College of Chiropractic-Real Centro Universitario Escorial-María Cristina, Paseo de los Alamillos, 2, 28200 San Lorenzo de El Escorial, Madrid Spain; 3 Molecular Imaging Unit, Spanish National Cancer Research Center (CNIO), Madrid, Spain; 4 Epidemiology and Biostatistics Unit (IIS-Fundación Jiménez Díaz), Madrid, Spain

**Keywords:** Prognostic markers, Osteoarthritis

## Abstract

Osteoarthritis (OA) is a degenerative disease characterized by injury of all joint tissues. Our previous study showed that in experimental osteoporosis, chiropractic manipulation (CM) exerts protective effects on bone. We here assessed whether CM might ameliorate OA by improving subchondral bone sclerosis, cartilage integrity and synovitis. Male New-Zealand rabbits underwent knee surgery to induce OA by anterior cruciate ligament injury. CM was performed using the chiropractic instrument ActivatorV 3 times/week for 8 weeks as follows: force 2 setting was applied to the tibial tubercle of the rabbit right hind limb (TM-OA), whereas the corresponding left hind limb received a false manipulation (FM-OA) consisting of ActivatorV firing in the air and slightly touching the tibial tubercle. After sacrifice, subchondral bone integrity was assessed in the tibiae by microCT and histology. Cartilage damage and synovitis were estimated by Mankin’s and Krenn’s scores, respectively, and histological techniques. Bone mineral density and content in both cortical and trabecular compartments of subchondral bone decreased in OA rabbits compared to controls, but partially reversed in the TM-OA group. Trabecular bone parameters in the latter group also showed a significant improvement compared to FM-OA group. Moreover RANKL, OPG, ALP and TRAP protein expression in subchondral bone significantly decreased in TM-OA rabbits with respect to FM-OA group. CM was associated with lower Mankin’s and Krenn’s scores and macrophage infiltrate together with a decreased protein expression of pro-inflammatory, fibrotic and angiogenic factors, in TM-OA rabbits with respect to FM-OA. Our results suggest that CM may mitigate OA progression by improving subchondral bone as well as cartilage and synovial membrane status.

## Introduction

Osteoarthritis (OA) is one of the most common chronic diseases affecting all anatomical structures of the joint, namely cartilage, subchondral bone and synovial membrane^1^. This disease affects about 15% of the population aged 25–75 years, and its prevalence significantly increases with age, affecting 70% of the population over 65 years^2^.

Although OA has been described as a cartilage disorder, changes in the underlying (subchondral) bone also occur in this disease^3^. In this sense, different molecular alterations associated with the latter bone remodeling, e.g., in expression of nuclear factor ligand receptor kappa B (RANKL) and osteoprotegerin (OPG), have been described in OA^4–7^.

Preclinical and clinical studies point to the observed alterations in subchondral bone as an important OA pathogenic factor^8^. In fact, studies in animal models of combined osteoporosis (OP) and OA (OPOA) demonstrate that OP induces cartilage damage^9^. In this setting, the observed significant correlation between deterioration of subchondral bone and cartilage injury indicates that alterations in subchondral microstructure aggravate cartilage damage^10^.

Currently, no effective pharmacotherapy is available for OA, and the treatment of OA patients is based on established guidelines for structural conservation of the joints by correcting postures and avoiding joint overloads^11^. Likewise, good physical activity is recommended since mechanical stimulation can improve the initial stages of OA^11^.

Extracorporeal shock wave therapy (ESWT) has been described as a novel alternative for the treatment alternative in OA^12^. By mechanisms still poorly understood, application of shock waves appears to exert beneficial effects on both chondrocytes and subchondral bone remodeling^13^. However, the intensity of the shock waves applied as well as the duration and pattern of the treatment are variable, thus being difficult to analyze and compare the results obtained in different studies^14^. In fact, degenerative effects in joint tissue have been described when using ESWT intensity^15^. In this regard, low energy shock wave devices, such as ActivatorV Adjusting Instrument (Activator Methods International, Phoenix, AZ) used for chiropractic manipulation (CM)^16^ might be an alternative to ESWT in OA treatment. Compared to ESWT generators, the peak amplitude of the pressure waves generated by ActivatorV is 20-fold smaller^17^.

Previous data in cells cultures^17^ and synthetic blocks analogous to spinal tissues^18^ have demonstrated that the input force exerted by ActivatorV produces a maximum kinetic energy of 0.3 J; which is below the energy necessary to induce tissue damage^19^.

Recently, we reported that CM, using ActivatorV, induces an improvement in bone mineral density (BMD) and bone microarchitecture in an experimental rat model of OP^20^. Considering these previous findings and given the impact of subchondral bone in cartilage damage in OA, we hypothesized that ActivatorV-based CM might prevent the evolution of OA at least in part through the improvement of this bony tissue. In this study, we used male New-Zealand rabbits undergoing knee surgery to induce OA, as a well- characterized animal model^21^.

## Materials and methods

### Animals

Thirteen male New Zealand rabbits (12–13 weeks of age) (Granja San Bernardo, Pamplona, Spain), were included in the study. Rabbits were placed in cages under standard conditions (room temperature 20 ± 0.5 °C, relative humidity 55 ± 5%, and under 12 h/12 h light/dark photoperiod), given food and water ad libitum and allowed to move without restriction^22^.

### Animal procedures

After 2 weeks of adaptation to our facilities, OA was induced in both knees of each of ten rabbits by anterior cruciate ligament section^21^. The remaining three rabbits were used as healthy controls. The surgery was performed under general anesthesia (intramuscular administration of 20 mg/ml xylazine (Rompun, Bayer, Kiel, Germany) and 50 mg/ml ketamine (Ketolar, Pfizer, Hameln, Germany) at 3:1 ratio), under aseptic conditions in the operating room^22–24^.After 2 weeks of surgery, CM was performed using ActivatorV at setting 2 with preload of 3.705 lb/inch spring rate applied to the tibial tubercle of the rabbit right hind limb (true manipulation, TM) at a 90° angle from medial to lateral side^20,25^. The contralateral left hind limb received a false manipulation (FM) consisting of ActivatorV firing in the air and gently touching the tibial tubercle. These procedures were repeated 3 times/week for 8 weeks (Supplementary Fig. S1). At the end of the treatments, animals were sacrificed by an intracardiac injection of 50 mg/kg pentobarbital (Tiobarbital, B. Braun Medical, Barcelona, Spain). Femoral condyles, tibial plateaus and synovial membranes were collected for further histological, immunohistochemical and Western blot analysis or microstructural studies.

### Microcomputerized tomography (microCT) analysis of rabbit tibiae

Left and right rabbit tibiae were scanned using a high-resolution microCT system (GE eXplore Locus lCT scanner, GE Healthcare, London, Canada), as previously described^20^.For subchondral cortical bone, regions of interest (ROIs) were analyzed, starting in the cortical bone edge until the appearance of the first trabeculae, corresponding to approximately 20 slides per sample. For subchondral trabecular bone, a characteristic region in medial posterolateral position was selected^26^. The reconstructed images of both subchondral cortical and trabecular bone were analyzed using MicroView software, version 2.2 with Advanced Bone Analysis Plus (GE Healthcare, London, Canada)^20^. Both BMD and bone mineral content (BMC) were evaluated in subchondral cortical and trabecular bone. Moreover, different trabecular parameters, namely bone volume/tissue volume (BV/TV), trabecular thickness (Tb. Th), trabecular number (Tb. N) and trabecular separation (Tb. S) were assessed^20^.

### Macroscopic tissue analysis

Degenerative changes of medial and lateral femoral condyles in the rabbit cartilage were analyzed macroscopically and classified into four grades: 0 = intact surface, 1 = irregular surface; 2 = surface fibrillation, 3 = erosion according to the modified Laverty’s grading system^27^.

### Cartilage histopathological alterations

After microCT was performed, left and right tibiae were decalcified with 10% EDTA, pH 7.7, solution for 4 months. Then, the tibae were cleaved in a sagittal plane along the central portion of the articular surface of each medial tibial plate corresponding to the weight-bearing area, and subsequently embedded in paraffin. Five-µm tibial sections were stained with safranin O-fast green for histological analysis as previously described^23^. Stained sections were evaluated by two independent observers using a modified Mankinʼs grading score system which analyses four different parameters with a total score up to 21: structure (0–8), proteoglycan staining (0–6), loss of chondrocytes (0–4), and clone formation (0–3)^28^. The final score of each sample was the average of two independent scores.

### Histological synovitis grading

Synovial membranes from both knees of each rabbit were sectioned (5 µm) and stained with hematoxylin and eosin. Synovitis was evaluated according to the Krenn’s score^29^, assessing lining hyperplasia, activation of synovial stroma related to fibrosis, and tissue infiltration. Each item was evaluated by two blind observers using a subscale of 0–3 points, where 0 indicated absence, 1 mild, 2 intermediate and 3 strong evidence of synovitis, as previously described^24^. The total score was obtained from the sum of partial grades with a maximum total score of 9^24,29^.

### Immunohistochemical localization of RANKL, OPG and alkaline phosphataseALP

The distribution pattern of cells expressing RANKL, OPG and ALP was also assessed in paraffin-embedded tibia sections. Briefly, after deparaffination, sections were rehydrated in graded ethanol and incubated in 4% bovine serum albumin (BSA) and 3% sheep serum to block unspecific immunobinding, based on Pérez-Baos et al.^24^. Mouse monoclonal antibodies against RANKL, ALP (Santa Cruz Biotech, Santa Cruz, CA, USA) or OPG (R&D Systems, Minneapolis, MN, USA) (at 1:200 dilution) were added overnight, at 4 °C. This was followed by incubation with corresponding biotinylated goat anti-mouse IgG (GE Healthcare, Little Chalfont, Buckinghamshire, UK), at 1:200 dilution, and peroxidase ABC with 3,3′-diaminobenzidine tetra-hydrochloride as chromogen (Dako, Golstrup, Denmark). Sections were counterstained with hematoxylin, mounted in DPX medium (VWR International, Leuven, Belgium) and photographed using an automated iScan Coreo slide scanner (Ventana Medical Systems, Oro Valley, AR, USA) as previously described^24^. Three random areas per slide were selected blinded to group assignment and quantified with Image J software^24^. Results are expressed as a percentage of positive stained area in relation to the total tissue area. The negative controls involved incubation with an IgG isotype.

### Tartrate-resistant acid phosphatase (TRAP) staining

TRAP staining was performed as previously described^30^. In brief, after deparaffination and 0.1 M acetate buffer, pH 5, washing, sections were incubated with TRAP buffer consisting of:0.1 M acetate buffer pH 5, 0.3 M sodium tartrate, naphtol AS-MX phosphate (10 mg/ml), 0.1% Triton X-100 and fast red violet LB staining reagent (0.3 mg/ml) for 30 min^30^. Samples were counterstained with fast green reagent for 45 s, and mounted in DPX (VWR Inter-national Ltd, Leuven, Belgium). TRAP-positive cells with three or more nuclei were counted as multinucleated osteoclasts in 5 random high-power fields (400×, bone area 0.04 mm^2^) per sample in the subchondral bone of each rabbit^31^. Total osteoclasts were expressed as the mean of TRAP positive cells per mm^2^ in each experimental group.

### Immunohistochemistry of rabbit macrophages and endothelial cells in synovial membrane

Macrophages were identified in the synovial membrane, using a mouse monoclonal anti-rabbit macrophage antibody (RAM11; Dako, Glostrup, Denmark), according to a previously described protocol^32^; whereas endothelial cells were identified with mouse monoclonal CD31 antibody (Abcam, Cambridge, UK), as reported^33^. To evaluate both RAM-11 and CD31 positive immune reactivity, sections were photographed using an automated iScan Coreo slide scanner and five random areas per slide were selected, blinded to group assignment, and quantified with Image J software, as previously reported^24,34^. The results were expressed as percentage of positive area in relation to the total tissue area. An IgG isotype was used as negative control.

### Western blot

Proteins from synovial membranes were extracted and processed as described elsewhere^32,35,36^. Briefly, tissue proteins were extracted with mechanical disintegration of tissue in RIPA buffer, pH 8 (Sigma-Aldrich, St. Louis, MO, USA), supplemented with protease inhibitor cocktail P8340 (Sigma-Aldrich) and phosphatase inhibitor cocktail Set II (Calbiochem, La Jolla, CA, USA). Protein content was determined by bicinchoninic acid (BCA) (Thermo Fisher Scientific, Rockford, IL, USA). Protein extracts (25–60 µg) were separated on 10–15% polyacrylamide-SDS gels under reducing conditions. After electrophoresis, samples were transferred onto nitrocellulose membranes, followed by blocking with 3% BSA in 50 mM Tris–HCl, pH 7.6, and 150 mM NaCl with 0.05% Tween-20. After overnight incubation with mouse polyclonal anti-rabbit VEGF-164 (1:1,000, Abcam, Cambridge, UK), mouse monoclonal anti-rabbit MMP3 (1:1,000, Santa Cruz Biotechnology, Santa Cruz, CA, USA),guinea pig monoclonal anti-rabbit IL1-β (1:150, Cloud-Clone, Houston, TX), mouse polyclonal anti-rabbit TNFα (1:200, Cloud-Clone, Houston, TX, USA) and mouse monoclonal anti-rabbit COX-2 (1:1,000, Santa Cruz Biotechnology, Santa Cruz, CA, USA)at 4 °C; horseradish peroxidase-conjugated anti-mouse IgG (GE Healthcare, Little Chalfont, Buckinghamshire, UK), or anti-guinea pig IgG (Abcam, Cambridge, UK) for appropriate primary antibodies was added for 1 h, at room temperature. As loading control, mouse monoclonal anti β-actin antibody (Santa Cruz Biotechnology, Santa Cruz, CA, USA) was used. Protein bands were developed with Luminata Crescendo Western HRP detection (Millipore, Billerica, MA, USA), and analyzed by densitometric scanning using Amersham Imager 600 (GE Healthcare Life Sciences, London, Canada).

### Statistical analysis

Results were expressed as median and interquartile range (IQR) throughout the text. We used non-parametric Kruskal–Wallis test with a post-hoc correction (Dunn’s procedure) for comparison between multiple groups, and Mann–Whitney U test for comparison between two groups. Each limb was analyzed as an independent sample for the studies. P < 0.05 was considered significant. Statistical analysis was performed using GraphPad Prism V 5.01 software (GraphPad, La Jolla, CA, USA).

### Ethical approval

Animal procedures and experimental protocols followed the guidelines of European Union directives (2010/63/EU), Spanish regulation (RD 53/2013), as well as the Community of Madrid Administration (PROEX REF. 135/16), and were approved by the Animal Research Committee at the IIS-Fundación Jiménez Díaz.

## Results

### CM improves microstructural parameters of subchondral bone in OA rabbits

A significant loss of subchondral bone mass was observed in the tibia of the OA group of rabbits compared to control animals at time of sacrifice, as confirmed by microCT. Thus, both BMD and BMC in cortical and trabecular compartments of subchondral bone were lower in FM-OA rabbits than in control rabbits (Table 1). ActivatorV adjustment (TM-OA group) produced an increase in BMD and BMC in both skeletal compartments, without reaching the corresponding values in control animals (Table 1). In addition, in subchondral trabecular tibia of FM-OA rabbits, we found a significant decrease in BV/TV as well as in Tb.N, Tb.S and Tb. Th values, compared to those in healthy controls, which was partially reversed by CM (TM-OA group) (Table 1).

**Table 1:** Changes in subchondral cortical and trabecular bone measured by microCT.

Parameters	Control (n = 6)	FM-OA (n = 10)	TM-OA (n = 10)
**Subchondral cortical bone**			
BMD (g/cm^3^)	5.25 (0.57)	4.64 (0.81)*	4.96 (0.74)^#,^*
BMC (g)	0.0075 (0.001)	0.0051 (0.004)*	0.0068 (0.003)^#,^*
**Subchondral trabecular bone**			
BMD (g/cm^3^)	2.61 (0.27)	2.16 (0.29)**	2.42 (0.30)^#,^*
BMC (g)	0.044 (0.006)	0.035 (0.004)**	0.042 (0.006)^#,^*
BV/TV (%)	0.321 (0.027)	0.169 (0.021)*	0.274 (0.095)^#,^*
Tb. Th (mm)	0.172 (0.010)	0.146 (0.019)**	0.137 (0.009)^#,^***
Tb. N (mm^−1^)	1.848 (0.352)	1.189 (0.168)*	2.009 (0.479)^#^
Tb. Sp (mm)	0.314 (0.136)	0.699 (0.102)*	0.362 (0.139)^#,^*

Analysis by microCT of the subchondral cortical and trabecular compartments of subchondral bone in the metaphysis of proximal tibiae of healthy and OA rabbits with false manipulation (FM-OA) or true manipulation (TM-OA). Values are median (IQR). *p < 0.05, **p < 0.01, ***p < 0.001 vs healthy control, ^#^p0.05 vs FM-OA. P values were obtained using non-parametric Kruskal–Wallis test with a post-hoc correction (Dunn’s procedure) for comparison among multiple groups, and Mann–Whitney U test for comparison among two groups.

### CM counteracts the increase of subchondral bone remodelling in OA rabbits

**Figure 1:**
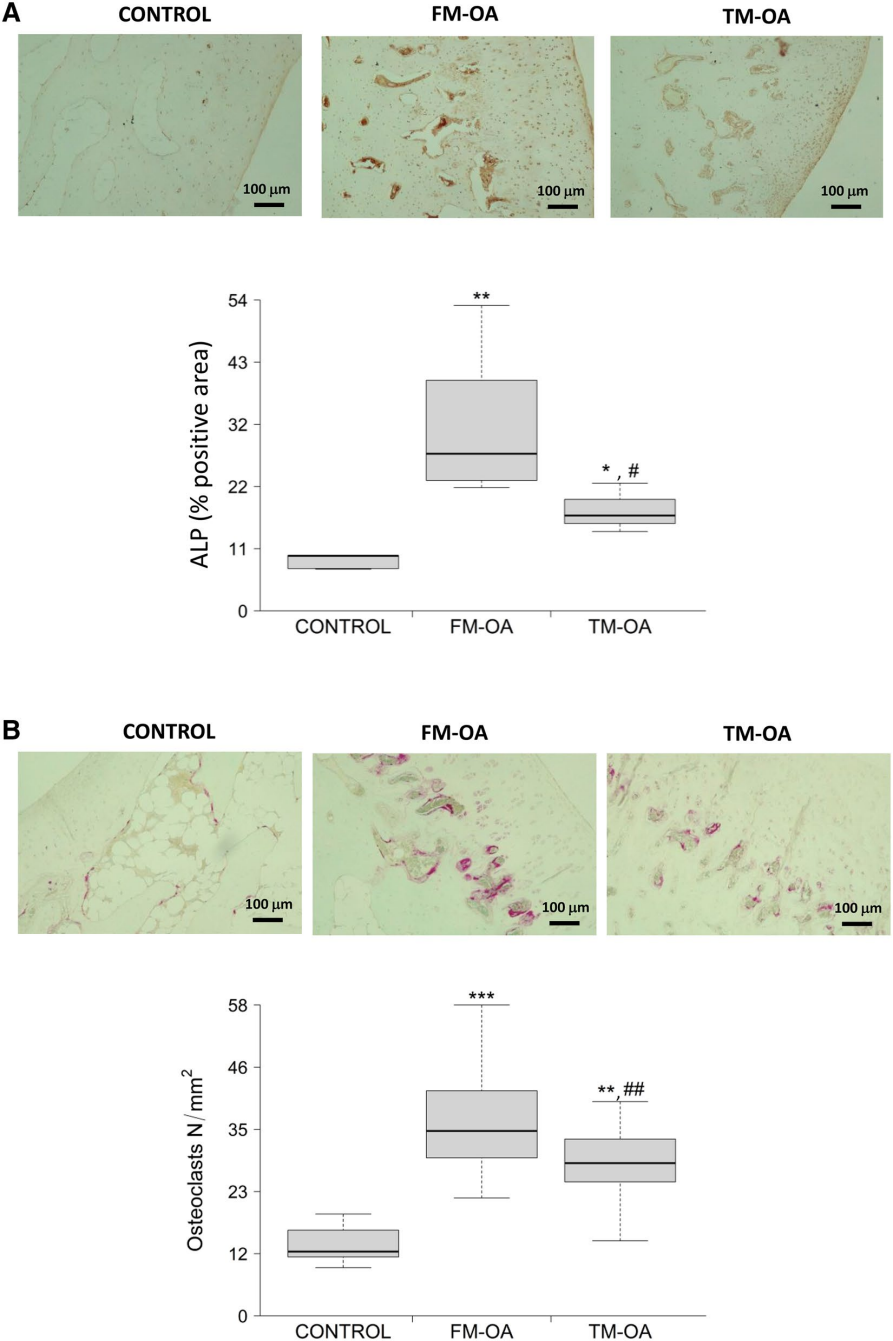
Immunohistochemical evaluation of ALP and TRAP in rabbit subchondral tibia. Immunohistochemistry of ALP (**A**) and TRAP (**B**) was performed onparaffin-embedded samples of the subchondral tibia from each group of rabbits as described in the text. Magnification, ×10, scale: 100 µm. Densitometry values of each type of immunostaining are also shown. Values are median (IQR) (Control, n = 6; FM-OA, n = 10; and TM-OA, n = 10). *p < 0.05, **p < 0.01, ***p < 0.001 vs Control; ^#^p < 0.05, ^##^p < 0.01 vs FM-OA. P values were obtained using non-parametric Kruskal–Wallis test with a post-hoc correction (Dunn’s procedure) for comparison among multiple groups, and Mann–Whitney U test for comparison between two groups.

Immunostaining of ALP, a bone formation marker, increased significantly in the subchondral tibia of FM-OA rabbits, with regards to that in healthy controls, but it significantly decreased-without reaching normal values- in the TM-OA group (Fig. 1A and Supplementary Table 1). Moreover, a similar trend of changes in the number of subchondral bone osteoclasts, as determined by TRAP staining, was revealed in these three groups of rabbits (Fig. 1B and Supplementary Table 1).

**Figure 2:**
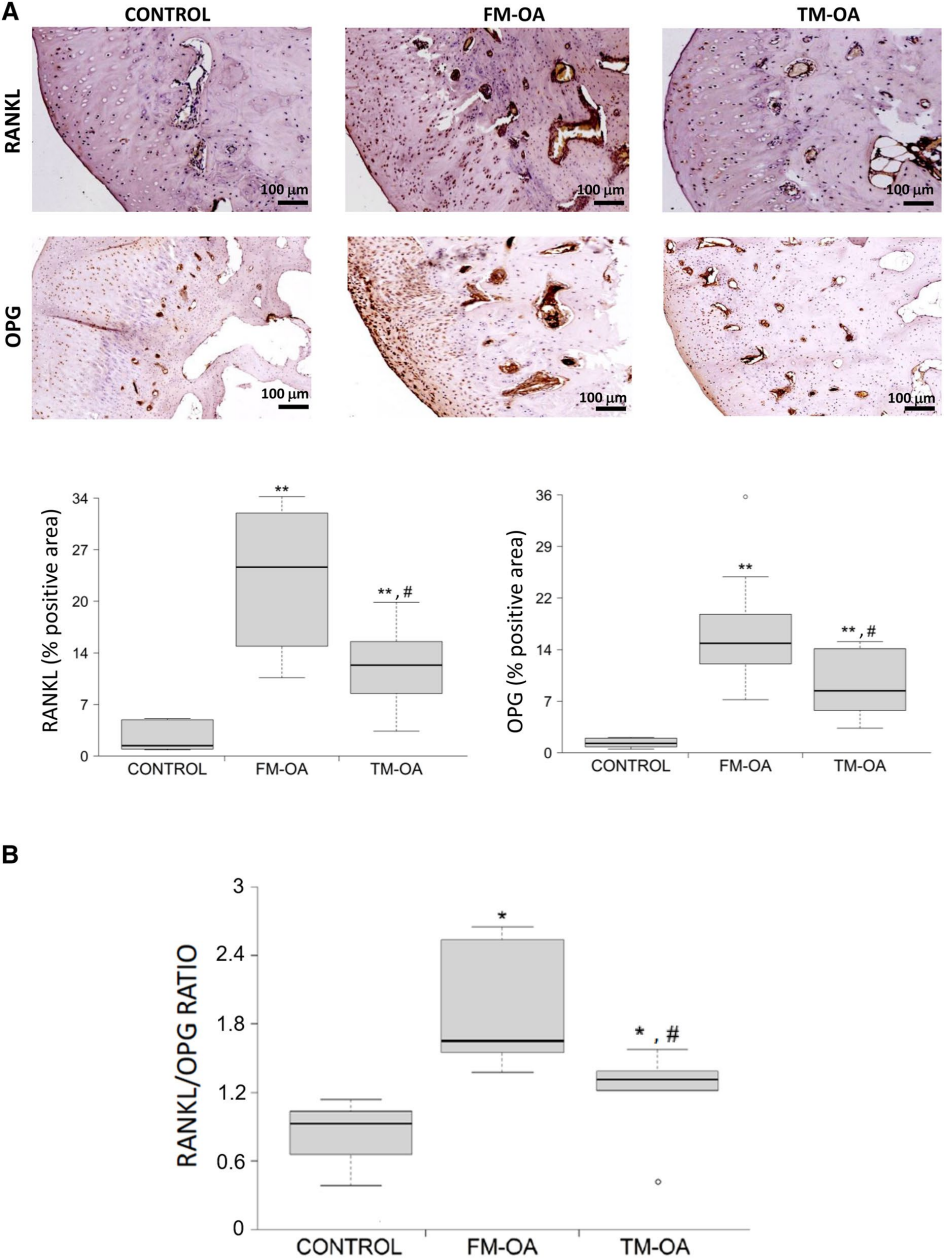
Immunohistochemical analysis of RANKL and OPG in rabbit subchondral tibia. (**A**) Immunohistochemistry of RANKL and OPG was performed on paraffin-embedded samples of the subchondral tibia from each group of rabbits as described in the text. Magnification, × 10, scale: 100 µm. Corresponding densitometry values are also shown. (**B**) RANKL/OPG positivity ratio in the subchondral bone of each rabbit group studied. Values are median (IQR) (Control, n = 6; FM-OA, n = 10; and TM-OA, n = 10). *p < 0.05, **p < 0.01 vs Control; ^#^p < 0.05 vs FM-OA. P values were obtained using non-parametric Kruskal–Wallis test with a post-hoc correction (Dunn’s procedure) for comparison among multiple groups, and Mann–Whitney U test for comparison between two groups.

Since the RANKL/OPG system has a key role in bone remodelling^37^, and it has previously been found to be dysregulated in the subchondral bone of OA rabbits^10,11^, we here explored possible changes in this system induced by CM in our experimental OA model. In the subchondral bone of the tibiae of FM-OA animals, a significant increase in immunostaining for both RANKL and OPG was observed compared to that of healthy rabbits, but it was lower for both factors in the TM-OA group (Fig. 2A and Supplementary Table 1). As a consequence, a significant increase of the RANKL/OPG ratio was observed in the FM-OA rabbit subcondral tibiae, compared to that in healthy controls, which partially normalized in the TM-OA group (Fig. 2B and Supplementary Table 1).

### CM modifies cartilage and synovial membrane damage in OA rabbits

**Figure 3:**
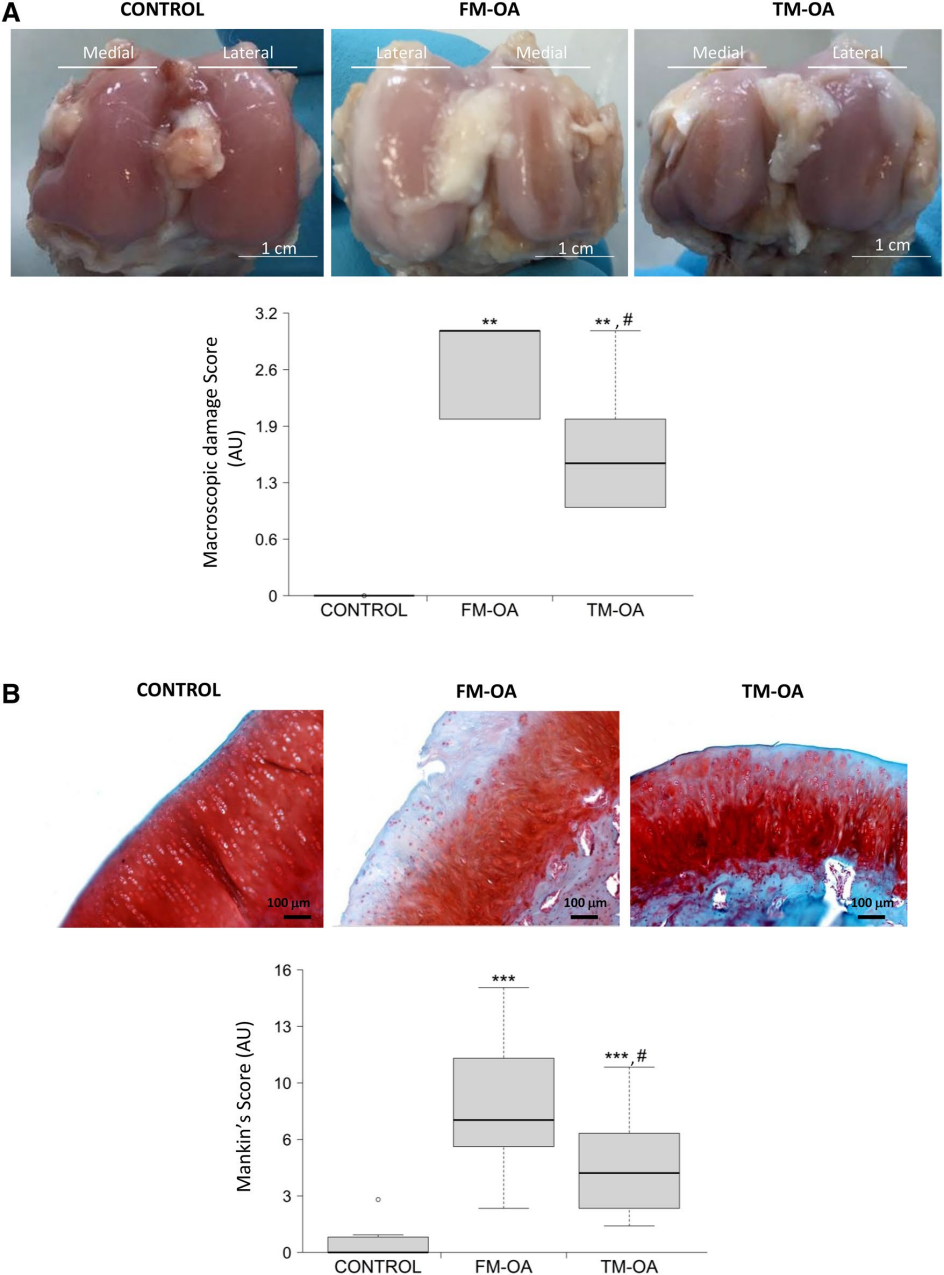
Macroscopic and histological analysis of rabbit cartilage. (**A**) Representative images of femoral cartilages of each group tested. The erosive aspect and brilliance, as well as the presence of osteophytes or other irregularities were assessed in the cartilage tissue. The medial and lateral condyles of each femur areindicated. (**B**) Tibia samples were immunostained with safranin and evaluated by Mankin’s score. Damage was evaluated according to the following parameters: proteoglycan loss (by safranin staining), tissue erosion, chondrocytes organization or clone formation, and vascular infiltrate. Mankin’s values are shown in the corresponding bar graphic. Values are median (IQR) (Control, n = 6; FM-OA, n = 10; and TM-OA, n = 10). *p < 0.05, **p < 0.01, ***p < 0.001 vs control; ^#^p < 0.05 vs FM-OA. P values were obtained using non-parametric Kruskal–Wallis test with a post-hoc correction (Dunn’s procedure) for comparison among multiple groups, and Mann–Whitney U test for comparison between two groups.

We next assessed whether CM was able to modify cartilage damage in this rabbit model of OA, using a macroscopical grading in femoral condyles. Morphological changes such as discoloration due to loss of proteoglycan matrix and erosion were visible in the medial and lateral femoral condyles in OA rabbits, being more pronounced in the medial condyle (Fig. 3A and Supplementary Table 1). However, the severity of this damage was significantly lower in the TM-OA group, compared to the FM-OA group (Fig. 3A and Supplementary Table 1). Furthermore, tibial cartilage degradation was evaluated by Mankinʼs grading score. As expected, OA rabbit tibiae showed higher Mankinʼs scores than control rabbits, but this increase was less pronounced in the TM-OA group (Fig. 3B and Supplementary Table 1).

**Figure 4:**
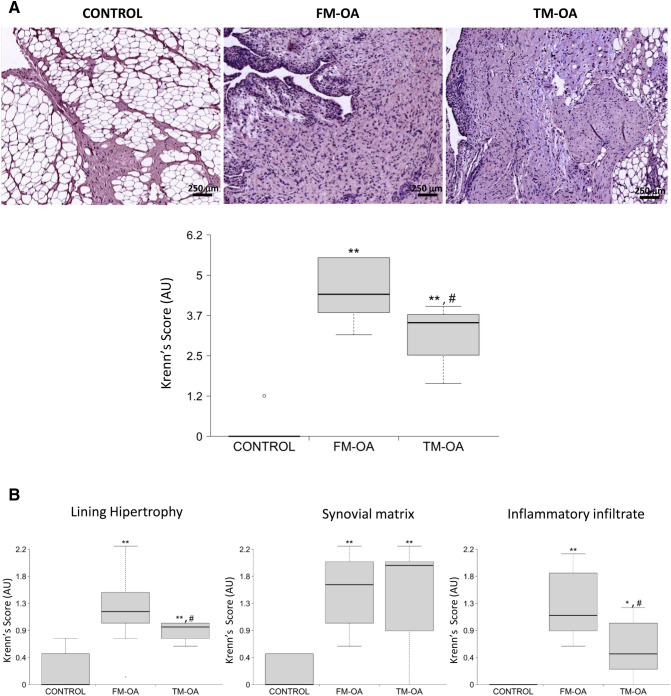
Synovitis grading in rabbit synovial membrane. (**A**) Representative sections of synovium stained with hematoxylin and eosin. Magnification: × 4, scale: 250 µm. Global synovitis score quantification according to Krenn’s score is shown. (**B**) Changes in different parameters analyzed separately according to Krenn’s score. Values are median (IQR) (Control, n = 6; FM-OA, n = 10; and TM-OA, n = 10). *p < 0.05, **p < 0.01 vs control; ^#^p < 0.05 vs FM-OA. P values were obtained using non-parametric Kruskal–Wallis test with a post-hoc correction (Dunn’s procedure) for comparison among multiple groups, and Mann–Whitney U test for comparison between two groups.

The Krennʼs score analysis revealed characteristic lesions in the OA rabbit synovial membrane, including lining thickening, an increment in stromal cellularity and presence of infiltrating cells in the synovial stroma (Fig. 4). Interestingly, the FM-OA group showed a significantly higher Krennʼ score than the TM-OA group (Fig. 4A and Supplementary Table 1), mainly at the expense of a decrease in inflammatory cells in the latter group (Fig. 4B and Supplementary Table 1).

### CM affects macrophage infiltration in synovial membrane of OA rabbits

**Figure 5:**
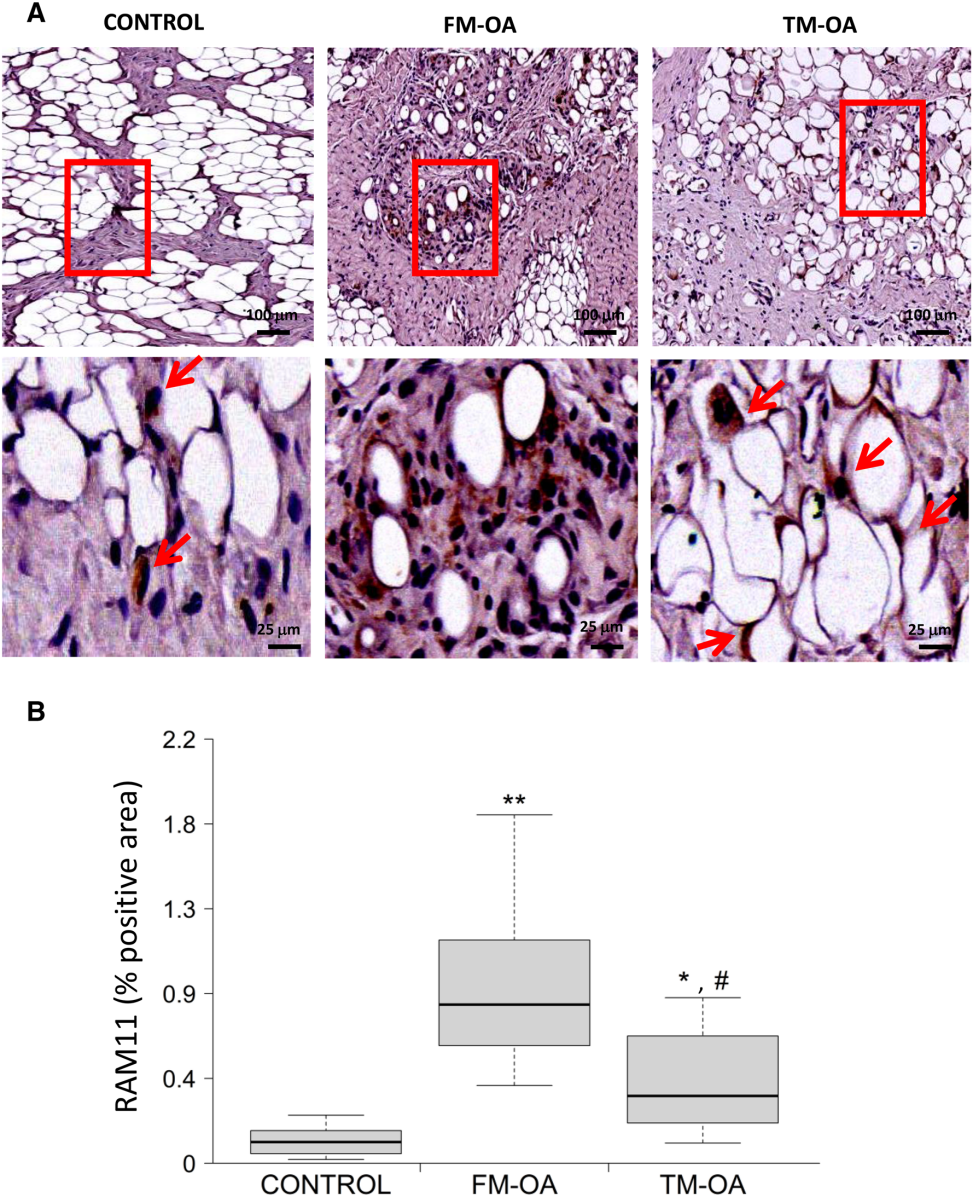
Evaluation of macrophage infiltrate in the rabbit synovial membrane. (**A**) Immunohistochemistry of representative synovium sections from each rabbit group studied, using anti-rabbit macrophage antibody RAM11. Magnification: ×10 (top images), ×40 (bottom images). Scale: 100 µm (top images), 25 µm (bottom images). (**B**) Corresponding densitometric analysis of RAM11 staining in the synovium of each group of animals. Values are median (IQR) (Control, n = 6; FM-OA, n = 10; and TM-OA, n = 10).*p < 0.05, **p < 0.01 vs control; ^#^p < 0.05 vs FM-OA. P values were obtained using non-parametric Kruskal–Wallis test with a post-hoc correction (Dunn’s procedure) for comparison among multiple groups, and Mann–Whitney U test for comparison between two groups.

Inflammatory infiltration was further confirmed by assessing the presence of macrophages in synovial membrane of OA rabbits. In contrast to healthy controls, FM-OA rabbits showed distinct and abundant RAM11-positive cells in both the synovial stroma and intimal layer (Fig. 5A and Supplementary Table 1). However, this RAM11 positivity in the synovial membrane significantly decreased in the TM-OA group, although it was still significantly higher than in healthy rabbits (Fig. 5B and Supplementary Table 1).

**Figure 6:**
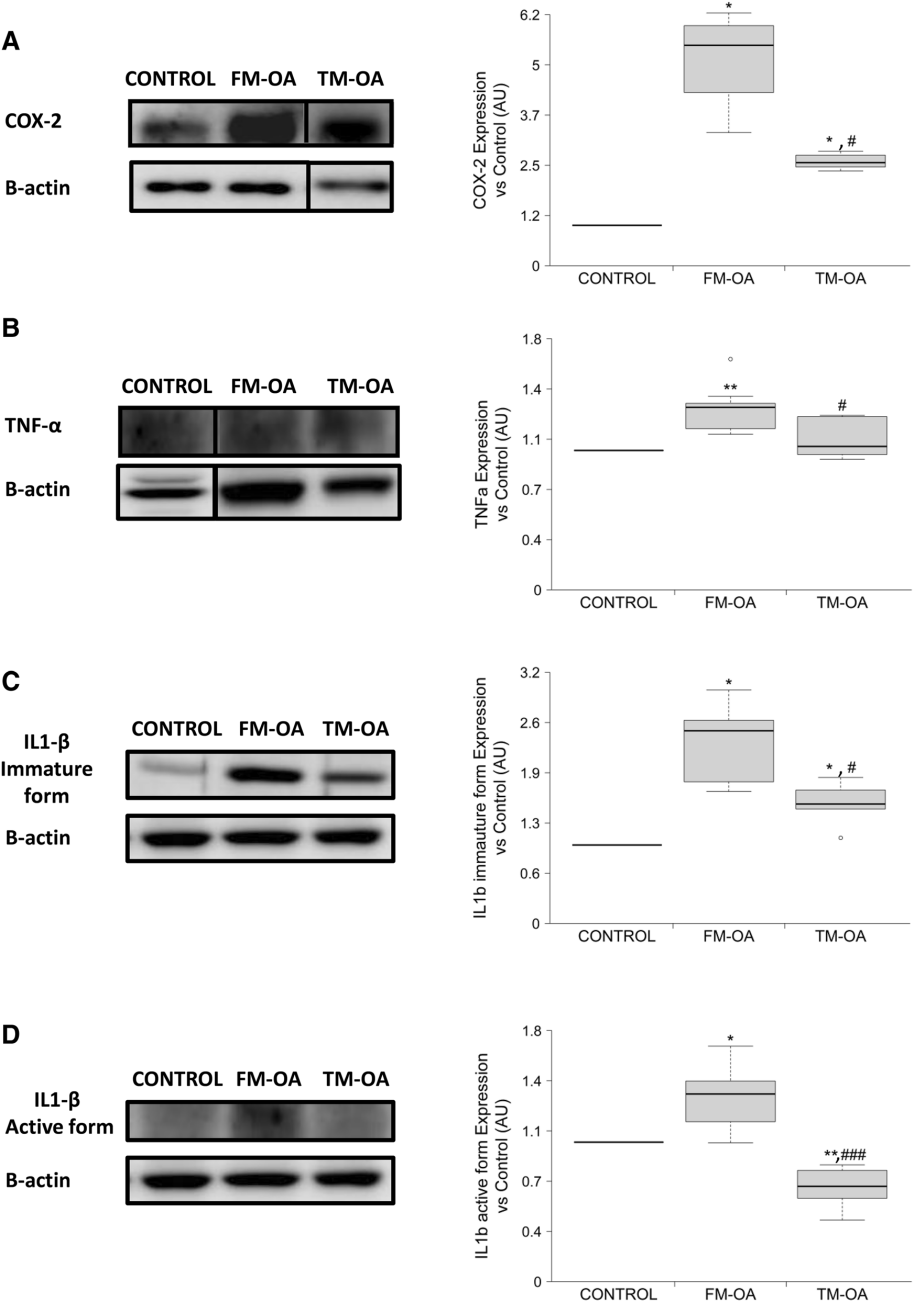
Presence of several proinflammatory mediators in rabbit synovial membrane. COX-2 (**A**), TNFα (**B**), and immature (**C**) and active (**D**) IL1-β protein expression were analyzed by Western blot in synovial membrane protein extracts from each experimental group studied. The dividing line (**A**,**B**) indicates that the samples derive from the same experiment and that gels/blots were processed in parallel. For simplicity, full-length blots are presented separately in Supplementary Fig. S2. Data are relative intensities of each protein signal normalized to that of β-actin for each experimental group, compared to the corresponding value in control (expressed as n-fold). Values are median (IQR) (Control, n = 6; FM-OA, n = 10; and TM-OA, n = 10). *p < 0.05, **p < 0.01 vs Control; ^#^p < 0.05, ^###^p < 0.001 vs FM-OA. P values were obtained using non-parametric Kruskal–Wallis test with a post-hoc correction (Dunn’s procedure) for comparison among multiple groups, and Mann–Whitney U test for comparison between two groups.

We next explored whether CM might be able to modify the expression of pro-inflammatory cytokines, namely IL1-β, TNFα and COX-2, in the synovial membrane of OA rabbits. Using Western blot analysis, OA was found to induce a marked increase of all these pro-inflammatory mediators in comparison to those in control animals, but this increase was significantly attenuated in the TM-OA group (Fig. 6, Supplementary Fig. S2 and Supplementary Table 1).

### CM effects on synovial fibrosis and angiogenesis of OA rabbits

**Figure 7:**
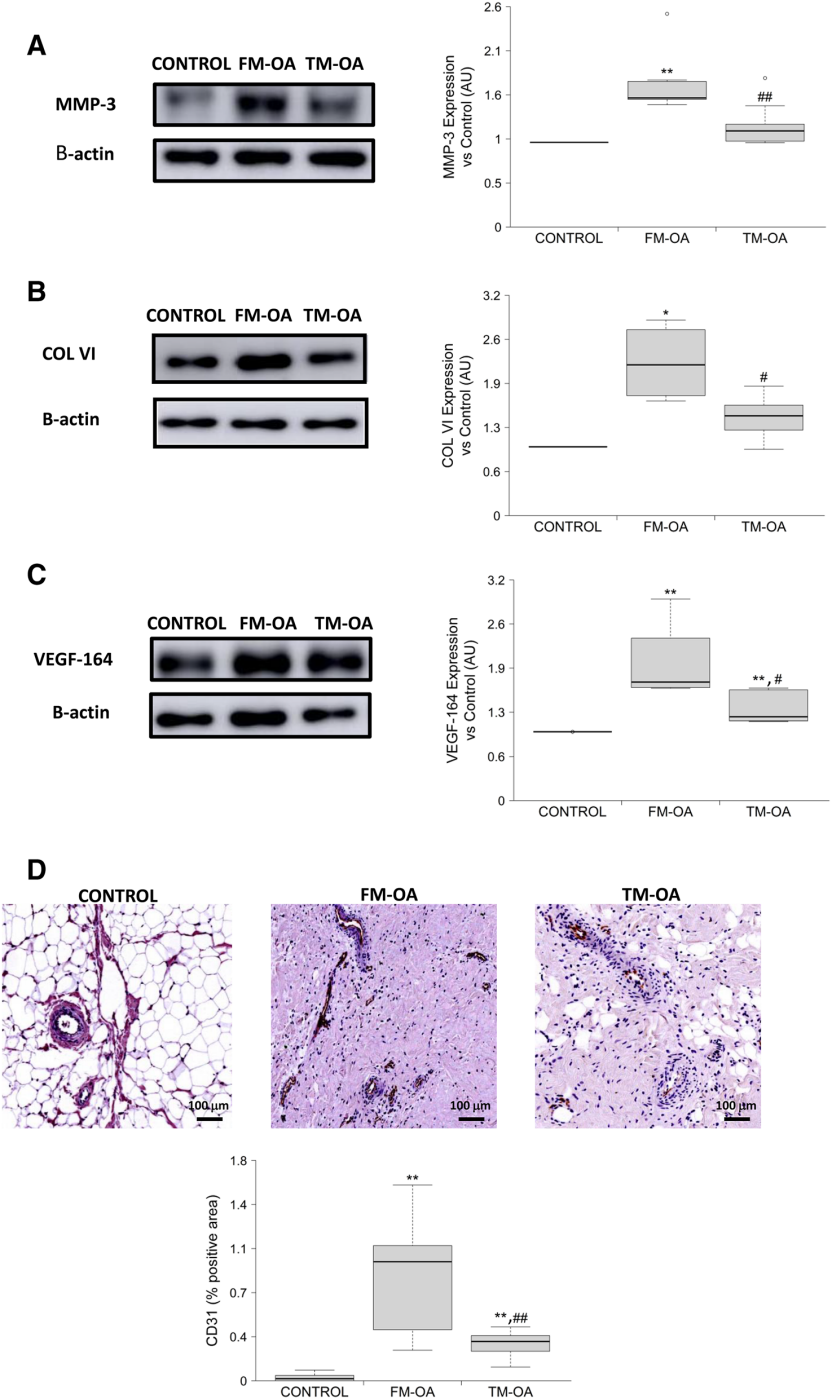
Protein expression of fibrotic and angiogenic markers in rabbit synovial membrane. MMP-3 (**A**), COL VI (**B**) and VEGF-164 (**C**) protein expression in synovial membranes from each animal group of rabbits studied in synovial protein extracts by Western blot. For simplicity, full-length blots are presented separately in Supplementary Fig. S3. Data are relative intensities of the protein signal normalized to that of β-actin for each experimental group, compared to the corresponding value in control (expressed as n-fold). (**D**) Immunohistochemistry of CD31 in representative synovium sections from each rabbit group studied. Magnification: ×10, scale: 100 µm. Quantitative results of corresponding densitometric analysis are shown in the bar graphic. Values are median (IQR) (Control, n = 6; FM-OA, n = 10; and TM-OA, n = 10) *p < 0.05, **p < 0.01 vs Control; ^#^p < 0.05, ^##^p < 0.01 vs FM-OA. P values were obtained using non-parametric Kruskal–Wallis test with a post-hoc correction (Dunn’s procedure) for comparison among multiple groups, and Mann–Whitney U test for comparison between two groups.

We also investigated the putative effects of CM on extracellular matrix formation and remodelling in synovial membrane of OA rabbits. We found an enhanced protein expression of MMP-3 and Col VI in OA animals compared to the control group, which was significantly lower in the TM-OA group than in the FM-OA group (Fig. 7A,B, Supplementary Fig. S3 and Supplementary Table 1).

Inflammation and angiogenesis in the synovium are closely integrated processes in the pathogenesis of OA progression^38^. In OA rabbits, we found that synovial membranes exhibited an increased protein expression of VEGF—an angiogenic factor—in comparison to that in control rabbits; this increase was significantly attenuated in the TM-OA group (Fig. 7C, Supplementary Fig. S3 and Supplementary Table 1). Moreover, the presence of vessels in the synovial tissue of OA rabbits was studied by CD31 immunostaining. A significant increase of CD31-positivity was found to occur in both OA groups studied, compared to that in control animals, but it was more dramatic in FM-OA rabbits (Fig. 7D and Supplementary Table 1).

## Discussion

The anterior cruciate ligament transection model is a well-known model in OA research^39^. The joint destabilization induced by this procedure leads to a rapid and severe degradation of articular cartilage, and also to subchondral bone changes^39^. In the present study, the observed positive effects of CM in this OA model in rabbits seem to support the association between subchondral bone remodeling and cartilage tissue integrity. Moreover, CM positively affected several OA-related changes in the synovial membrane, including inflammation and angiogenesis and extracellular matrix remodeling, in this animal model.

In this rabbit model, demineralization of the subchondral bone associated with OA was found to be reversed by CM, in agreement with our previous findings in a rat osteoporosis model^20^. Consistent results have previously been reported by using EWST in postmenopausal women with lumbar and femoral OP^40^. In the latter study, it was hypothesized that EWST stimulation would induce the expression of osteogenic growth factors, enhancing the activity of osteoblasts and/or inhibiting the differentiation of osteoclasts and bone resorption^40^. In this regard, the present study shows a CM-related increase in ALP, an osteoblastic marker, but a reduction of TRAP-positive osteoclasts together with a decreased RANKL/OPG ratio in subchondral bone of the TM-OA group as compared to the FM-OA group. Of note, the latter ratio has been suggested to be a possible target in OA therapy^41^. Our findings in this OA model thus suggest that the beneficial effects of CM on subchondral bone remodeling can be exerted by affecting both osteoblasts and osteoclasts.

In the present OA model, subchondral bone microarchitecture was also positively affected by CM as shown when comparing TM-OA and FM-OA groups at this tissue level. Specifically, an increase in Tb.N and a reduction in Th.S in the trabecular compartment were observed in the former group compared to the latter group of rabbits. This was consistent with our previous findings in trabecular bone of osteoporotic rats undergoing a similar ActivatorV treatment^20^. In contrast to the aforementioned study in rats, though, in the present OA model we also observed a decreased Tb.Th in the subchondral tibiae of FM-OA rabbits, which failed to improve in the TM-OA group. However, Tb.Th is a parameter that tends to be overestimated in rats^42,43^. The apparent inefficiency of CM on affecting Tb.Th in our present OA model might be related to the fact that a large voxel size in the case of the rabbit tibia determines image pixelation, which imposes uncertainty when measuring the binarized surface thickness^43^. Moreover, the anatomical differences between the rabbit and rat tibia is another factor which might explain the differences in the CM-induced changes observed in trabecular bone structure by microCT between our earlier report and the present study^44^. In any case, the observed increase in the number of trabeculae induced by CM in OA rabbits could compensate the reduction in their thickness, producing a general improvement in the mechanical properties of the subchondral bone in this setting. In this regard, some studies have reported that subchondral bone provides structural support to the overlying articular cartilage and plays an important role in the development of cartilage lesions associated to OA^1^. Indeed, the improvement of subchondral bone following administration of anti-resorptive agents, namely bisphosphonates, has been shown to prevent cartilage damage progression in OA^45^. In the present rabbit model, the positive effects of CM on subchondral bone were associated with less cartilage deterioration, both at macroscopic level in the femur and by Mankin’s score in the tibia, in OA rabbits. This chondroprotective effect associated with ActivatorV treatment is consistent with previous data using ESWT in rats^46^.

The true mechanisms whereby CM could exert these observed beneficial effects on damaged cartilage are unknown. Likely candidates as signaling molecules for a cross-talk between subchondral bone and articular cartilage in OA include bone remodeling factors targeting chondrocytes^47^. A previous study has suggested that the antiresorptive and chondroprotective effects of ESWT are a consequence of changes in mechanotransduction triggering different biological responses, such as anti-inflammatory processes, promotion of cell proliferation and neovascularization, thus favoring tissue regeneration and repair^48^. In addition, it has been speculated that the mechanical impulse generated by ActivatorV and the associated joint movement during spinal manipulative therapy could modulate the entry of sensory afferences (mechanoreceptors) to the central nervous system with subsequent modulation of muscle tension^19^. This muscular tension could achieve considerable articular reconditioning, relieving the overload to which the knee is exposed and thus, improving the status of the damaged cartilage in the context of OA.

The joint structure improvement triggered by CM in OA rabbits might be related to the parallel decreased synovitis, as shown by less macrophage infiltrate and lower levels of pro-inflammatory cytokines in the synovial membrane of these treated animals. In this regard, it has been proposed that components of the extracellular matrix might leak from the damaged cartilage into the synovial fluid and activate synovial macrophages^49^. This activation would lead to the release of proinflammatory cytokines and matrix metalloproteinases, generating a vicious circle of inflammation and cartilage breakdown^49^. In addition, recent data have shown that activated macrophages in the synovial membrane produce growth factors and chemokines promoting endothelial cell adhesion, and angiogenesis^50^. We here found that CM reduced neo-vascularization of synovial membrane associated with a reduction of inflammatory cells and an improvement of the deteriorated articular cartilage in OA rabbits. Recent studies have also shown that cytokines such as IL1-β and TNF-α produced by activated synoviocytes regulate the expression of metalloproteinases^51^. Our findings demonstrate that the observed elevated collagen VI and MMP-3 in the synovium of OA rabbits were significantly diminished by CM. As a note of interest in this respect, a recent study has shown that ESWT protected cartilage from biomechanical damage and prevented subchondral sclerosis through regulation of metalloproteinases in another model of OA^52^. Taken together, present data suggest that CM contributes to maintaining the synovial membrane structure through the combined reduction of metalloproteinases, angiogenesis and inflammatory infiltration, resulting in an improvement of the damaged joint in OA rabbits.

This study, however, presents some weaknesses and limitations. Thus, the results of the current study obtained in rabbits may not be translatable to humans due to the marked differences in joint biomechanics and gait of these animals compared to humans^53^. In addition, the number of animals used in this study was small, which may cause bias in statistical analysis and limits the statistical power in some analyzed parameters. In line with the above, further studies are strongly recommended to establish the role of CM in OA improvement as suggested by our present data.

In conclusion, the present study in rabbits suggests that CM may retard the progression of OA through an improvement of subchondral bone status and cartilage damage, associated with an ameliorated synovial damage.

## Supplementary information

Supplementary Information.
