# Supplementary material for: Beneficial effects of manually assisted chiropractic adjusting instrument in a rabbit model of osteoarthritis
Source: Sci Rep. 2020 Aug 6;10:13237. doi: 10.1038/s41598-020-70219-3 (PMC7413258; doi:10.1038/s41598-020-70219-3)
Supplement: Supplementary file 1 — Supplementary Information. [file 41598_2020_70219_MOESM1_ESM.pdf]

Beneficial effects of manually assisted chiropractic adjusting instrument in a rabbit model of osteoarthritis.

Conesa-Buendía FM[1], Mediero A[1], Fujikawa R[2], Esbrit P[1], Mulero F[3], Mahillo-Fernández I[4], Ortega-De Mues A[2].

[1]Bone and Joint Research Unit, Institute of Health Research (IIS-Fundación Jiménez Díaz), [2]Madrid College of Chiropractic-Real Centro Universitario Escorial-María Cristina, [3]Molecular Imaging Unit, Spanish National Cancer Research Center (CNIO), [4]Epidemiology and Biostatistics Unit (IIS-Fundación Jiménez Díaz), Madrid, Spain.

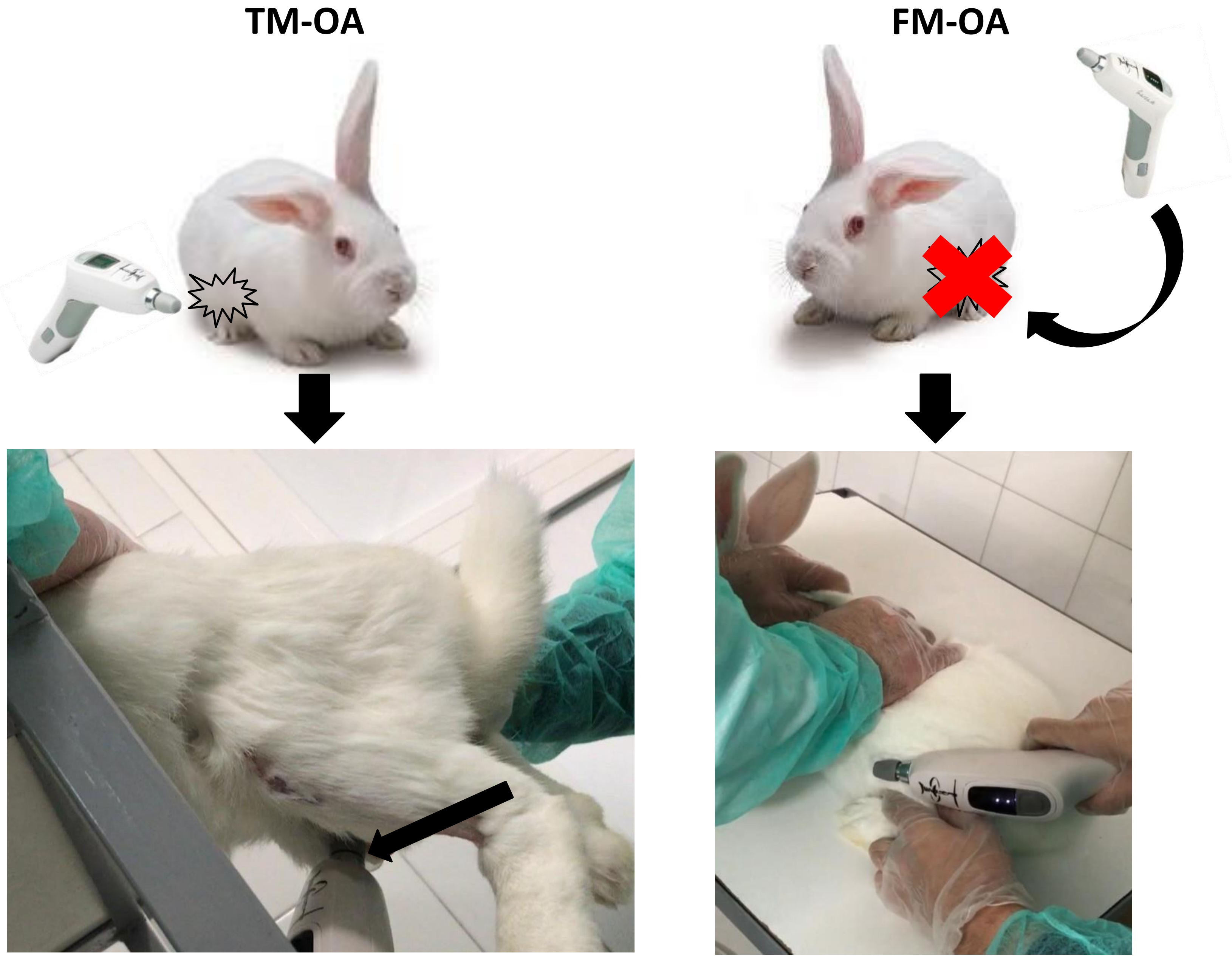

Supplementary Figure 1. Images of chiropractic manipulation (CM) carried out with ActivatorV in rabbits. On the left, the true manipulation (TM-OA) performed onto the tibial tubercle of the rabbit right hind limb at a 90° angle from medial to lateral side. On the right, the "false“ manipulation (FM-OA) consisting of ActivatorV firing and gently touching the left tibial tubercle.

Supplemmentary Figure 2. (A,C) Bands chosen to show in the Figure 6 the representative expression of (A) COX-2, (C) immature form of IL1-B and the corresponding loading controls obtained by Western blot for control, FM-OA and TM-OA conditions. (B, D) Order of the loaded samples in the gel and bands chosen to show in the Figure 6 the representative expression of (B) TNF- alpha, the active form of (D) IL 1 B and the corresponding loading controls analyzed by western blot for control, FM- OA and TM- OA conditions. The numbers correspond to different animals for each condition. (C control, FM false manipulation, TM true manipulation).

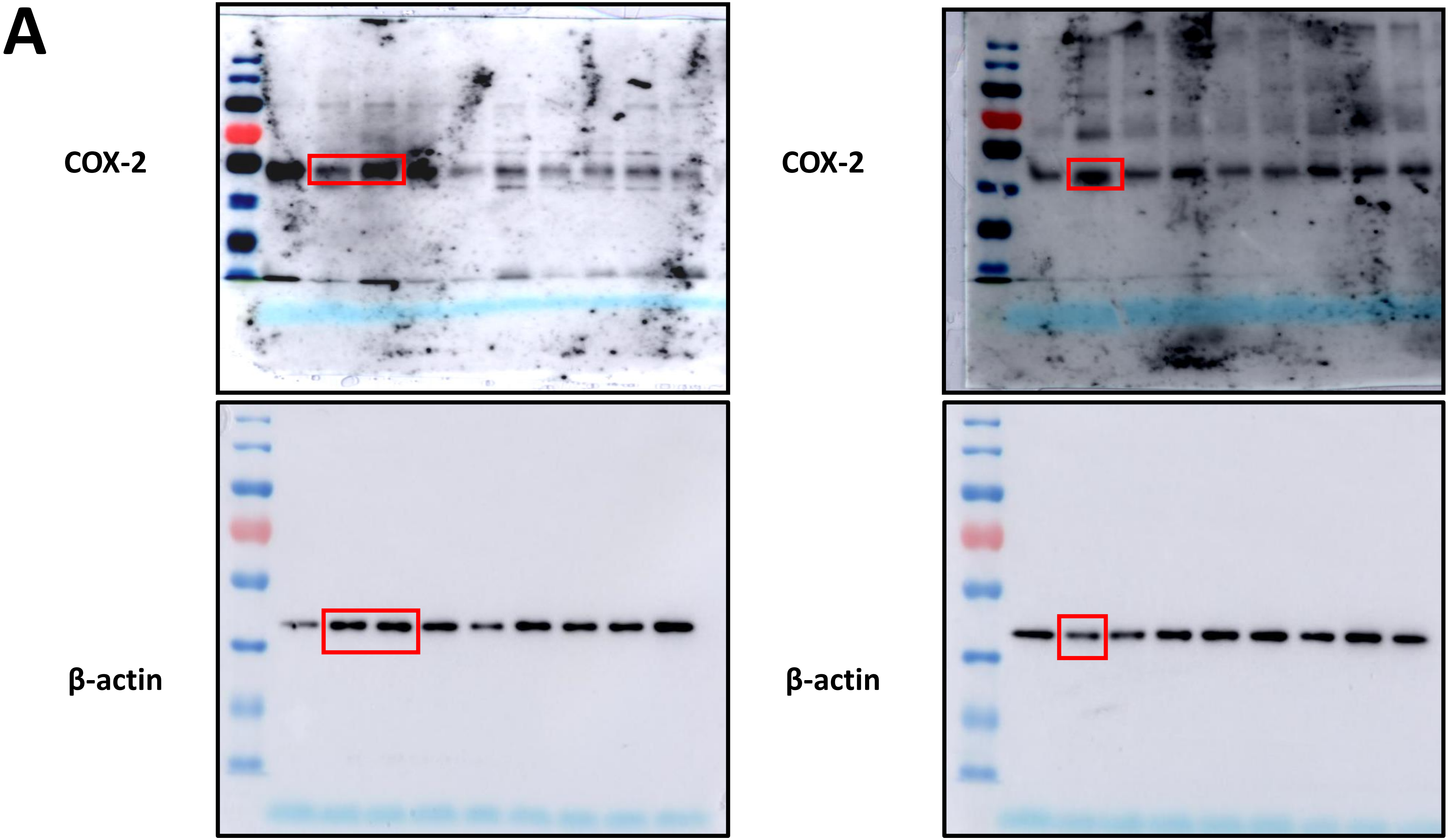

Supplementary Figure 2

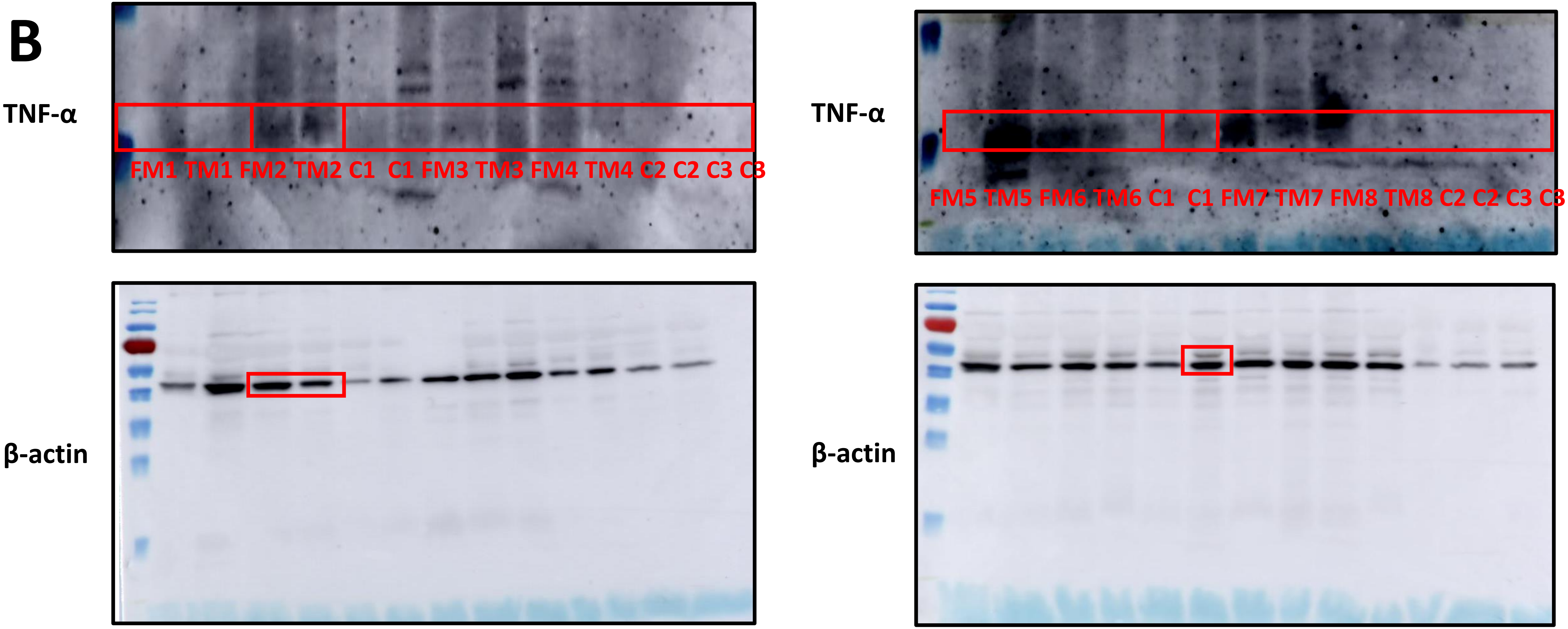

Supplementary Figure 2

C

IL1-β (immature form)

β-actin

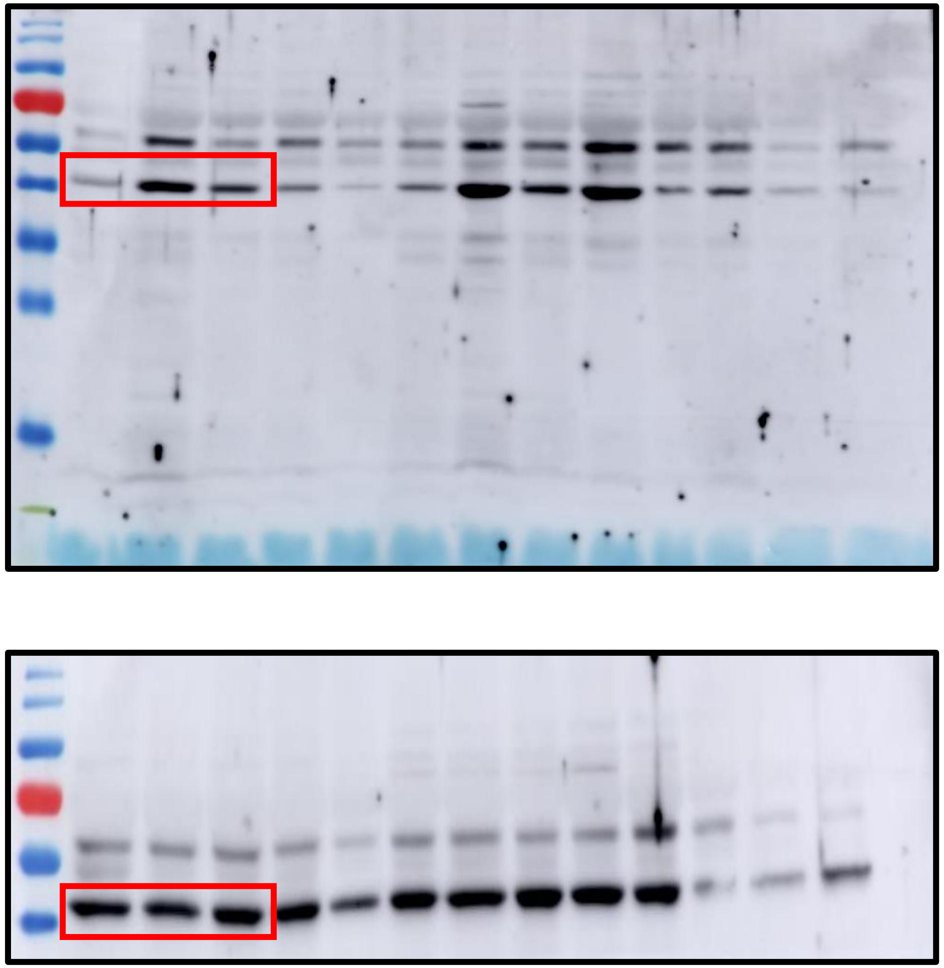

Supplementary Figure 2

D

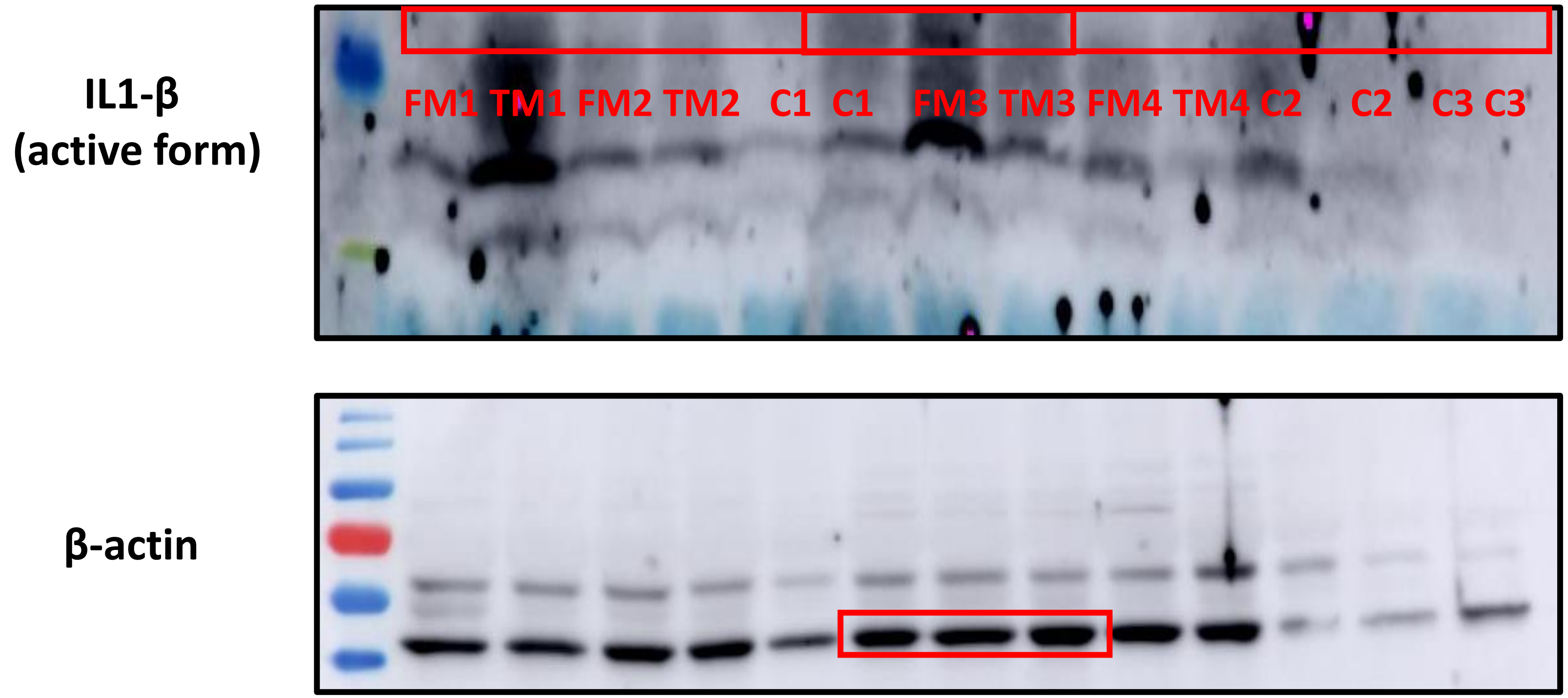

Supplementary Figure 2

Supplementary Figure 3. Bands chosen to show in the **Figure 7** the representative expression of **(A)** MMP-3, **(B)** COL VI, **(C)** VEGF-164 and the corresponding loading controls, obtained by Western blot for control, FM-OA and TM-OA conditions.

A

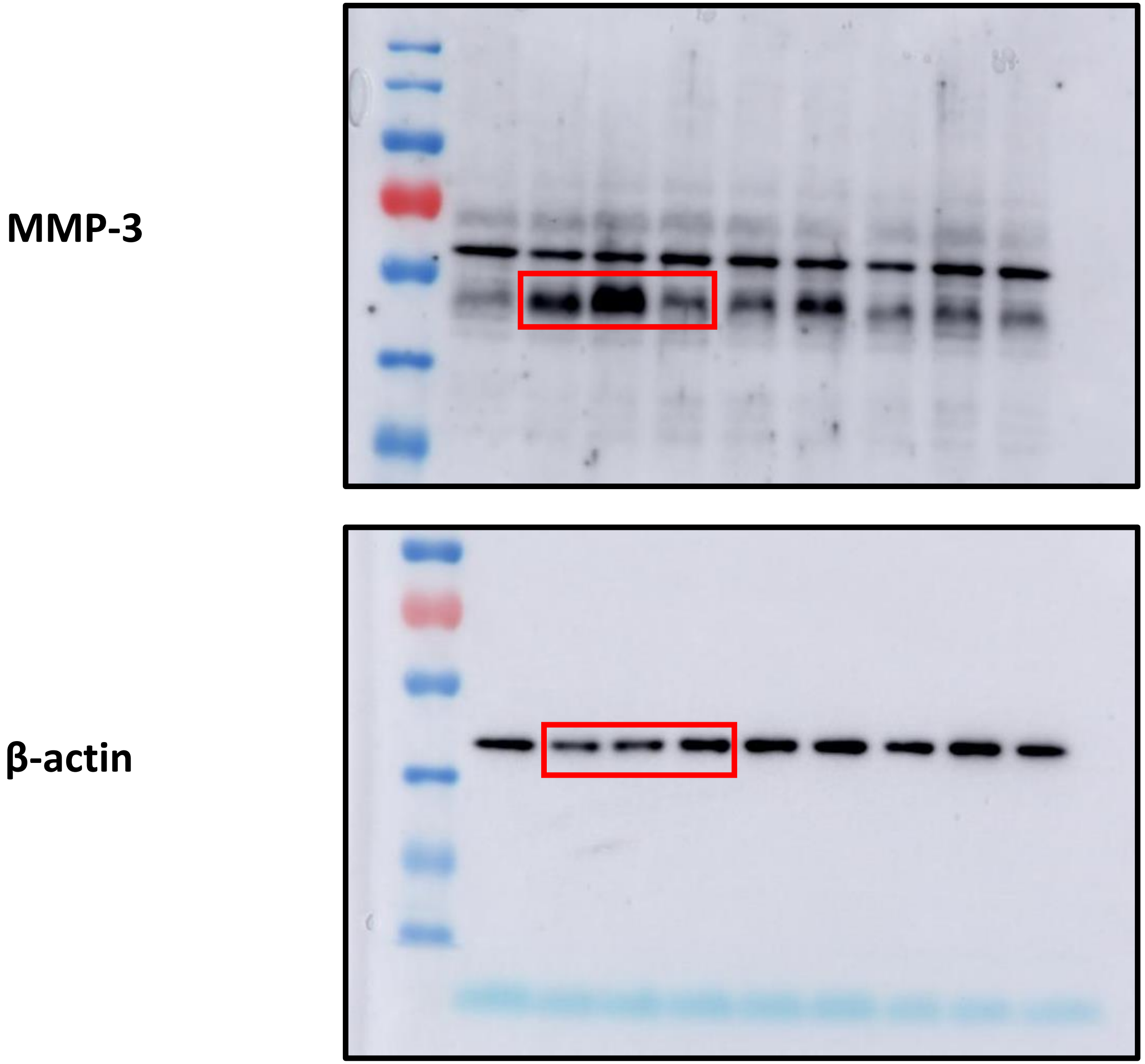

Supplementary Figure 3

B

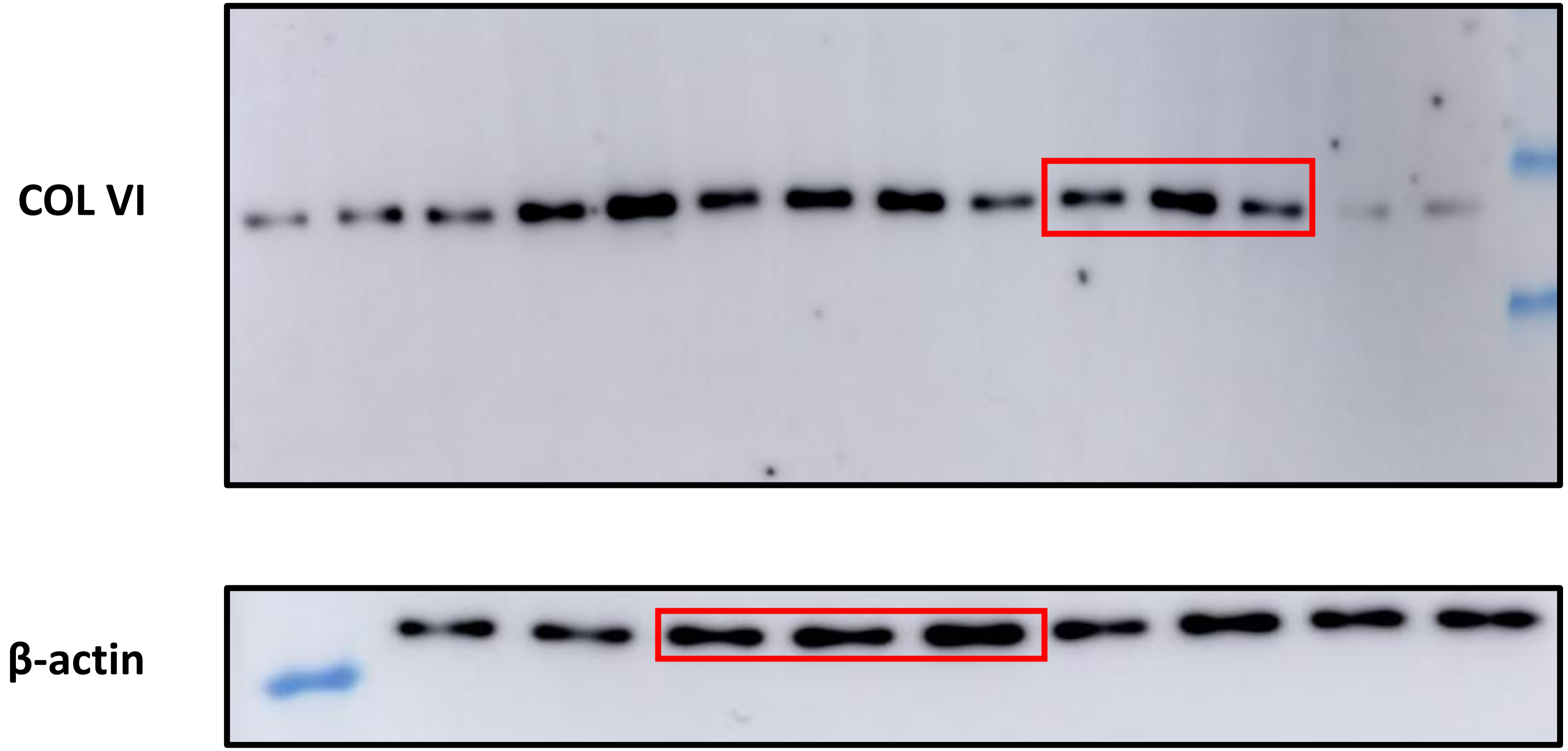

Supplementary Figure 3

C

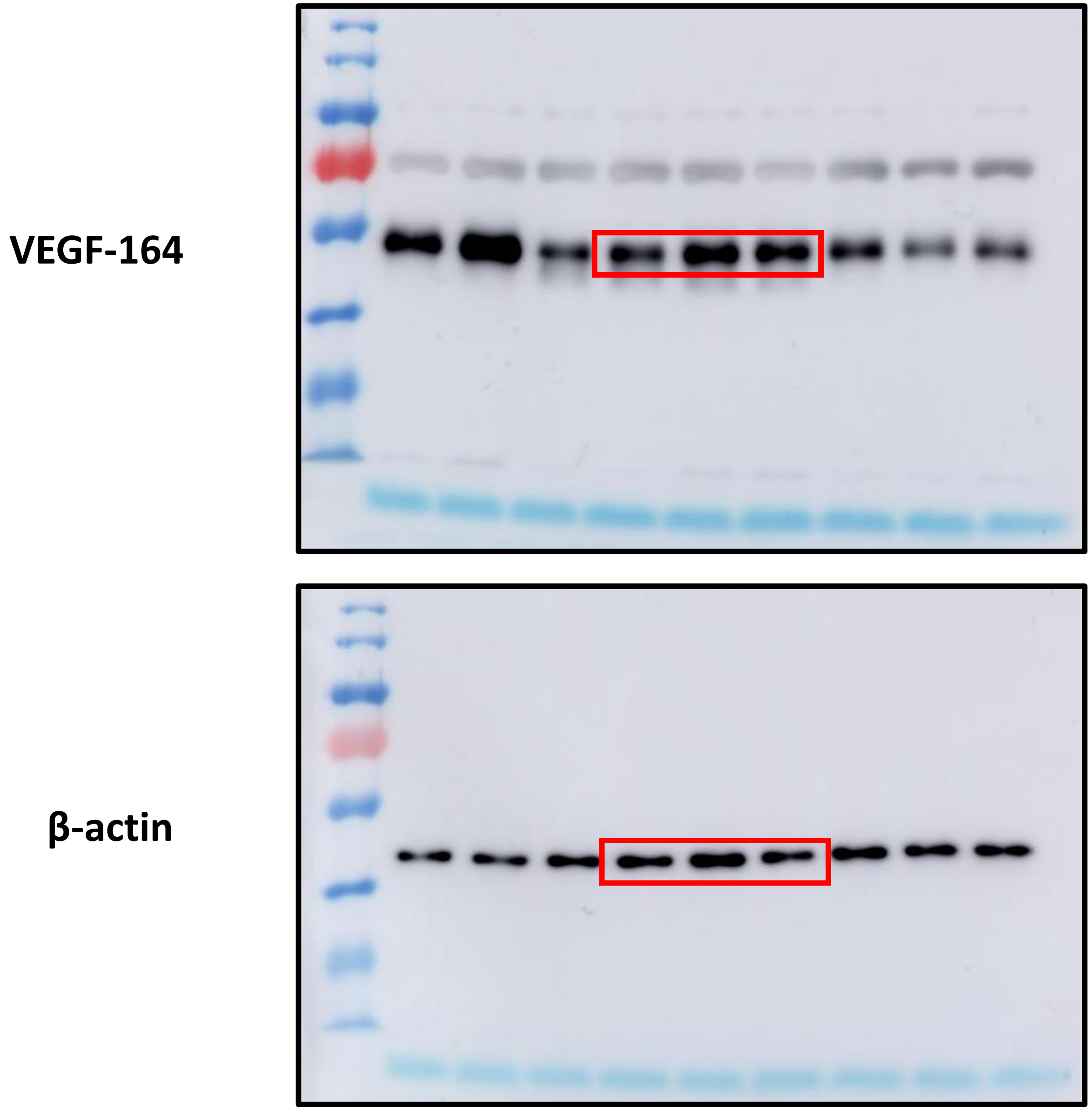

Supplementary Figure 3

Supplementary Table 1. Numerical data of the different parameters studied and presented as bar graphs in the text.

SUBCHONDRAL BONE			
Parameters	***CONTROL (n=6)***	***FM-OA (n=10)***	***TM-OA (n=10)***
ALP (% positive area)	9.6 (2.29)	27.3 (15.6)**	16.6 (4.20)*, #
Osteoclasts N/mm² ***(TRAP % positive area)***	12.0 (5.00)	34.5 (12.2)***	28.5 (7.75)**, ##
RANKL (% positive area)	1.36 (3.87)	24.9 (17.6)**	12.0 (6.08)**, #
OPG (% positive area)	1.31 (1.22)	15.3 (6.89)**	8.66 (8.22)**, #
RANKL/OPG Ratio	1.04 (0.48)	1.62 (0.78)*	1.39 (0.12) *, #
CARTILAGE			
Parameters	***CONTROL (n=6)***	***FM-OA (n=10)***	***TM-OA (n=10)***
Macroscopic damage Score (AU)	0.00 (0.00)	3.00 (1.00)**	1.50 (1.00)**, #
Mankin's Score (AU)	0.00 (0.81)	7.50 (4.50)***	4.50 (4.25)***, #
SYNOVIAL MEMBRANE			
Krenn' Score Total (AU)	0.00 (0.00)	4.38 (1.69)**	3.50 (1.25)**, #
Lining Hipertrophy (AU)	0.00 (0.38)	1.19 (0.44)**	0.94 (0.25)**, #
Synovial Matrix (AU)	0.00 (0.38)	1.62 (0.94)**	1.94 (0.91)**
Inflammatory Infiltrate (AU)	0.00 (0.00)	1.12 (0.78)**	0.50 (0.75)*, #
RAM11 (% positive area)	0.11 (0.10)	0.82 (0.55)**	0.35 (0.41)*, #
COX-2 Expression (AU)	1.00 (0.00)	5.45 (1.00)*	2.55 (0.27)*, #
TNFa Expression (AU)	1.00 (0.00)	1.31 (0.18)**	1.03 (0.27)#
IL1b Expression- Immature Form (AU)	1.00 (0.00)	2.46 (0.78)*	1.52 (0.24)*, #
Il1b Expression- Active Form (AU)	1.00 (0.00)	1.35 (0.29)*	0.68 (0.20)**, ###
MMP-3 Expression (AU)	1.00 (0.00)	1.52 (0.21)**	1.13 (0.20)##
COL VI Expression (AU)	1.00 (0.00)	2.19 (0.85) *	1.45 (0.35)#

VEGF-164 Expression (AU)	1.00 (0.00)	1.72 (0.43)**	1.21 (0.45)**, #
CD31 (% positive area)	0.02 (0.04)	0.97 (0.69)**	0.32 (0.13)**, ##

Values are median (IQR). *p<0.05, **p<0.01, ***p<0.001 *vs* healthy control, #p<0.05, ##p<0.01, ###p<0.001*vs* FM-OA. P values were obtained using non-parametric Kruskal-Wallis test with a post-hoc correction (Dunn's procedure) for comparison among multiple groups, and Mann-Whitney U test for comparison between two groups.
